# Implementing a mHealth intervention to increase colorectal cancer screening among high-risk cancer survivors treated with radiotherapy in the Childhood Cancer Survivor Study (CCSS)

**DOI:** 10.1186/s12913-022-08082-3

**Published:** 2022-05-23

**Authors:** Tara O. Henderson, Jenna K. Bardwell, Chaya S. Moskowitz, Aaron McDonald, Chris Vukadinovich, Helen Lam, Michael Curry, Kevin C. Oeffinger, Jennifer S. Ford, Elena B. Elkin, Paul C. Nathan, Gregory T. Armstrong, Karen Kim

**Affiliations:** 1 Department of Pediatrics, The University of Chicago, Chicago, IL USA; 2 Memorial Sloan Kettering Cancer Center, New York, NY USA; 3 St. Jude Children’s Research Hospital, Memphis, TN USA; 4 The University of Chicago, Chicago, IL USA; 5 Duke Cancer Institute, Durham, NC USA; 6 Hunter College, New York, NY USA; 7 Columbia University, New York, NY USA; 8 The Hospital for Sick Children, Toronto, Ontario Canada

**Keywords:** Colorectal cancer, cancer survivor, cancer prevention, cancer screening, Hybrid effectiveness-implementation design

## Abstract

**Background:**

Cancer survivors treated with any dose of radiation to the abdomen, pelvis, spine, or total body irradiation (TBI) are at increased risk for developing colorectal cancer (CRC) compared to the general population. Since earlier detection of CRC is strongly associated with improved survival, the Children’s Oncology Group (COG) Long-Term Follow-Up Guidelines recommend that these high-risk cancer survivors begin CRC screening via a colonoscopy or a multitarget stool DNA test at the age of 30 years or 5 years following the radiation treatment (whichever occurs last). However, only 37% (95% CI 34.1–39.9%) of high-risk survivors adhere to CRC surveillance. The **A**ctivating cancer **S**urvivors and their **P**rimary care providers (PCP) to **I**ncrease colo**re**ctal cancer **S**creening (ASPIRES) study is designed to assess the efficacy of an intervention to increase the rate of CRC screening among high-risk cancer survivors through interactive, educational text-messages and resources provided to participants, and CRC screening resources provided to their PCPs.

**Methods:**

ASPIRES is a three-arm, hybrid type II effectiveness and implementation study designed to simultaneously evaluate the efficacy of an intervention and assess the implementation process among participants in the Childhood Cancer Survivor Study (CCSS), a North American longitudinal cohort of childhood cancer survivors. The Control (C) arm participants receive electronic resources, participants in Treatment arm 1 receive electronic resources as well as interactive text messages, and participants in Treatment arm 2 receive electronic educational resources, interactive text messages, and their PCP’s receive faxed materials. We describe our plan to collect quantitative (questionnaires, medical records, study logs, CCSS data) and qualitative (semi-structured interviews) intervention outcome data as well as quantitative (questionnaires) and qualitative (interviews) data on the implementation process.

**Discussion:**

There is a critical need to increase the rate of CRC screening among high-risk cancer survivors. This hybrid effectiveness-implementation study will evaluate the effectiveness and implementation of an mHealth intervention consisting of interactive text-messages, electronic tools, and primary care provider resources. Findings from this research will advance CRC prevention efforts by enhancing understanding of the effectiveness of an mHealth intervention and highlighting factors that determine the successful implementation of this intervention within the high-risk cancer survivor population.

**Trial registration:**

This protocol was registered at clinicaltrials.gov (identifier NCT05084833) on October 20, 2021.

**Supplementary Information:**

The online version contains supplementary material available at 10.1186/s12913-022-08082-3.

## Background

### Cancer survivors and CRC risk

With the tremendous success in the treatment of childhood and adolescent cancer, there is a growing population of over 500,000 childhood cancer survivors in the US [1]. However, childhood cancer survivors are at risk for morbidity and premature mortality associated with their primary cancer and its treatment, with a 10.4 year loss of life expected compared to the general population [2–5]. While recurrence of the primary disease is the leading cause of premature mortality, subsequent malignant neoplasms (SMN) are the second leading cause among childhood cancer survivors. Focused efforts for early identification and prevention of these SMNs are needed to reduce mortality in this population.

A standardized incidence ratio (SIR), the ratio of the observed number of cases in a cohort compared to the expected number in the age-matched general population, indicates that childhood cancer survivors treated with abdominal or pelvic radiotherapy (RT) are almost four times (SIR 4.2, 95% CI 2.8–6.3) more likely to develop CRC compared to the general population, with their elevated risk evident by the age of 30 years with no plateau [6]. In fact, CRC represents the greatest absolute excess risk (AER) of all digestive subsequent primary neoplasms (SPNs) in childhood cancer survivors, with 11 excess colorectal cancers per 100,000 person-years (95% CI: 9–13) [7]. As in the general population, childhood cancer survivors usually develop screen-detectable adenomas prior to the development of a carcinoma [8, 9]. Since earlier detection of precancerous lesions (i.e., adenomas) is strongly associated with improved survival in the general population, screening with colonoscopy or a multitarget stool DNA test is recommended by the Children’s Oncology Group Long-Term Follow-Up Guidelines [10, 11] starting at age 30 years or 5 years following RT (whichever occurs last) in all survivors exposed to any dose of RT to the abdomen, pelvis, spine, or total body irradiation (TBI).

Prior work has shown that most survivors are unaware of: 1) their risk for SMN, including CRC; and 2) their recommended screening [12]. The majority of adult survivors of childhood cancer are no longer followed at a cancer center, and most primary care physicians (PCP) who care for survivors are similarly unaware of the risks or guidelines for CRC screening [13, 14]. Consequently, many survivors at elevated risk for CRC are not screened according to current recommendations, placing them at risk for preventable morbidity and premature mortality. Indeed, only 37% (95% CI 34.1–39.9%) of high-risk survivors adhere to CRC surveillance [15].

### Present study

This article describes a hybrid type II effectiveness and implementation study, entitled, “**A**ctivating cancer **S**urvivors and their **P**rimary care providers (PCP) to **I**ncrease colo**re**ctal cancer **S**creening” (ASPIRES). The intervention trial will simultaneously focus upon the effectiveness of an mHealth intervention for participant CRC screening completion (colonoscopy, negative multitarget stool DNA test, or positive multitarget stool DNA test + colonoscopy) and the evaluation of the implementation process using the Consolidated Framework for Implementation Research (CFIR) [16]. This article highlights the intervention design and study procedures related to conducting a hybrid effectiveness-implementation study targeting childhood cancer survivors.

To accomplish the study objectives, the project will utilize the infrastructure of the Childhood Cancer Survivor Study (CCSS), a 31-institution retrospective cohort that includes 25,655 5-year survivors of childhood cancer diagnosed and treated in the United States and Canada between 1970 and 1999. The CCSS Coordinating Center, located at St. Jude Children’s Research Hospital, is a pivotal partner in this project [17].

### Conceptual framework

The ASPIRES study is guided by the Patient Activation Model [18], which suggests that activated patients are better prepared to participate in self-management. Patient activation is the degree to which an individual understands they must play an active role in managing their own health and health care, and the extent to which they feel able to fulfill that role [19]. Individuals with higher levels of patient activation are more likely to undergo screening [20]. Activation involves four stages: (1) believing that taking an active role as a patient is important; (2) having the confidence and knowledge necessary to take action; (3) taking action to maintain and improve one’s health; and (4) staying the course even under stress [18].

Although the Patient Activation Model is a vital component of our conceptual model, we will include other important variables, many of which are from the Health Belief Model [21, 22] and the Transtheoretical Model [23–27]. These variables include benefits and barriers to screening; implementation intentions; perceived seriousness of susceptibility to CRC; and self-efficacy.

The ASPIRES study is enhanced by direct health care provider activation. PCP activation is designed to increase provider knowledge about the high-risk status of childhood cancer survivors and national screening recommendations, improve the motivation to discuss with, and order testing for, their patients, and facilitate effective information exchange and communication [28, 29]. We will examine specific factors (i.e., years in practice, location, knowledge) that may impact PCP activation.

### Objectives

The ASPIRES study will assess the effectiveness, cost-effectiveness, and implementation of an intervention to increase the rate of CRC screening (colonoscopy and/or multitarget stool DNA test) among high-risk cancer survivors through interactive, educational text-messages and electronic resources provided to participants, and CRC screening resources provided to their primary care providers. The study objectives are listed below:

#### Primary objective

Evaluate the difference in the proportion of patients who complete the colonoscopy or multitarget stool DNA test (multitarget stool DNA test plus colonoscopy if multitarget stool DNA test is positive) within 12 months of enrolling on this study; this will be measured via self-report questions in the patient questionnaire.

#### Secondary objectives

1. Evaluate the difference in the proportion of patients who complete the colonoscopy or multitarget stool DNA test (multitarget stool DNA test plus colonoscopy if multitarget stool DNA test is positive) within 12 months of enrolling on this study; this will be measured via medical record confirmation.
2. Explore differences in the rates of completion of the colonoscopy or multitarget stool DNA test (multitarget stool DNA test plus colonoscopy if multitarget stool DNA test is positive) between the two intervention arms.
3. Use the CFIR to evaluate the implementation process and identify potential barriers and facilitators to the uptake of the intervention.
4. Identify potential moderators and mediators of the uptake of CRC screening that may strengthen or weaken the effectiveness of the proposed intervention.
5. Estimate the costs and incremental cost-effectiveness of the proposed intervention.

## Methods

### Study design

We will use a hybrid type II effectiveness and implementation design [30], to study the intervention’s effectiveness while also identifying facilitators and barriers to the implementation of the intervention [31]. While the University of Chicago will manage the study, our partners will play key roles. Memorial Sloan Kettering Cancer Center collaborators will conduct much of the data analysis, Columbia University colleagues will lead the cost and cost-effectiveness analysis, and the CCSS Coordinating Center partners at St. Jude Children’s Research Hospital will lead participant recruitment, administer the electronic informed consent process (see Additional File 1), create a study website for participants within their previously established personal study portal, and implement a messaging plan to communicate key study components to participants.

**Fig. 1:**
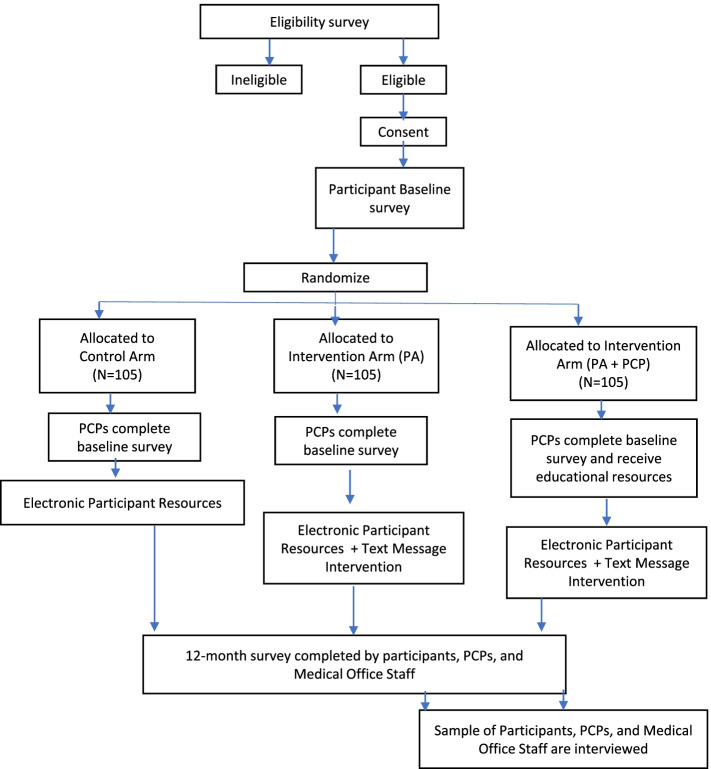
ASPIRES STUDY flow diagram

Participants will be randomized to one of three arms; the Best Practices Control (C) arm receives electronic educational resources (see Additional File 2) via the study website, the C + Patient Activation (PA) arm receives electronic educational resources via the study website as well as interactive text messages with links to videos and resources, and the C + PA + PCP Activation arm receives electronic educational resources via the study website, interactive text messages with links to videos and resources, and their PCPs are faxed educational materials (Fig. 1). For the purposes of this study, a PCP refers to a primary care provider or some other health care provider who the survivor sees regularly for care and who can order health screenings. Randomization will be stratified by age at study enrollment (30–44 and ≥ 45 years). We will use a permuted block randomization scheme with a random block size ranging from 6 to 12. The study principal investigators will be blinded to arm assignment.

Participants will be in the study for up to 15 months. All participants will complete a baseline and 12-month questionnaire via the CCSS Long-Term Follow Up study website (“My LTFU”). After completion of each of these questionanires, a participant will receive a $50 Amazon giftcard. A sample of 20 participants from each intervention arm will be invited to take part in an interview within 3 months after the completion of the 12-month questionnaire (see Implementation Outcome section below for more details). These interviewees will each receive a $50 Amazon giftcard. The primary care provider of each participant will complete a faxed baseline and 12-month questionnaire which include items to measure PCP activation, demographics, experience and comfort level providing care to cancer survivors, and knowledge about the healthcare needs of childhood cancer survivors; the 12-month questionnaire also includes CFIR items to aid in the evaluation of the implementation process. The PCPs will receive a $25 Amazon giftcard for completion of the baseline questionnaire, and a $50 Amazon giftcard for completion of the 12-month questionnaire. To gather additional data regarding the implementation process, we will also ask one medical office staff member associated with each participant’s PCP to complete a 12-month questionnaire via the phone. The medical office staff members will each receive a $25 Amazon giftcard for completion of the questionnaire. A sample of 20 primary care providers and 20 medial office staff from each intervention arm will take part in an interview within 3 months after the completion of the12-month questionnaire. Each interviewee will receive a $50 Amazon giftcard. We will identify each participant’s primary care provider by including a required section on the baseline questionnaire for participants to provide their PCP’s name and contact information. Similarly, the contact information for one medical office staff member working with each PCP will be collected via the baseline survey completed by each PCP. Participants without a PCP will be provided with a resource to encourage them to find a PCP, and will be asked repeatedly throughout the study to provide the study team with contact information for their PCP if one has been identified. The current study was approved by the University of Chicago Institutional Review Board (IRB20–1247), St. Jude Children’s Research Hospital Institutional Review Board (IRB21–0901), Memorial Sloan Kettering Cancer Center Institutional Review Board (IRB21–446), and the Columbia University Institutional Review Board (IRB-AAAT9278).

### Study participants

#### Eligibility

We plan to enroll 315 participants on the study. To confirm eligibility, participants will complete an eligibility survey. Participants must meet the following criteria in order to enroll on the study: 1) have previously enrolled in CCSS, 2) age > 30 years old, 3) were treated with radiation to the abdomen, pelvis, spine, or total body irradiation, 4) no history of CRC, 5) no family history of CRC, 6) have not had a colonoscopy in the last 5 years or multitarget stool DNA test in the last 3 years, 7) have a smartphone, 8) reside in the United States, and 9) speak English.

#### Recruitment

We will recruit participants from the CCSS; there are currently 1139 CCSS participants who fulfill the eligibility criteria (Table 1) [17]. The St. Jude CCSS Coordinating Center will recruit batches of 20–50 participants every few weeks. We will randomly sample from a list of eligible CCSS participants; we are planning to oversample racial minorites. Those who meet inclusion criteria will be emailed an invitation letter. After 2 weeks, a mailed letter will be sent to non-responders. A CCSS phone interviewer will call potentially eligible cohort members who fail to respond to two invitation letters and invite them to participate. We will continue attempting to accrue subjects from the eligibility list until 315 participants have been randomized into the study.

**Table 1:**
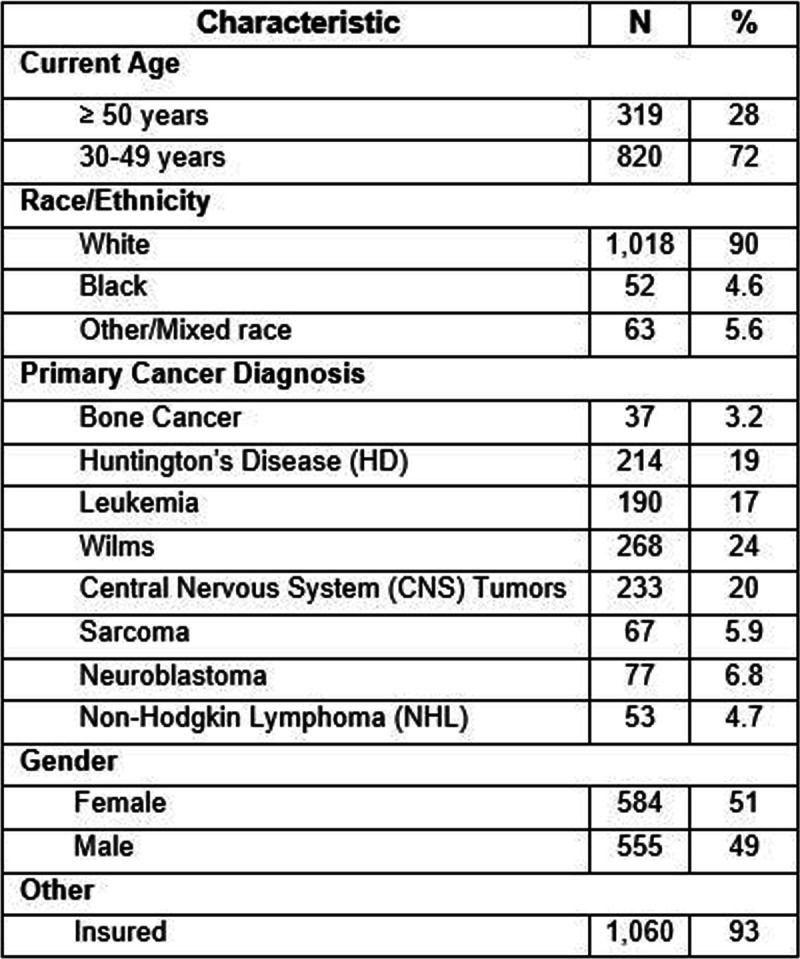
Characteristics of eligible CCSS participants (*n*=1139)

### Intervention

Figure 1 summarizes the intervention components that will be randomly assigned to each arm.

#### Electronic resources

The following downloadable, electronic resources were designed by the study team and will be available to all study participants via the study website (see Additional file 2):

- Survivorship Care Plan (SCP): an overview of past cancer treatment and screening recommendations personalized for the participant
- How to Use the SCP: a one-page tip sheet for using the SCP
- Potential Benefits and Other Considerations of Colorectal Cancer Screening: an overview of important factors to consider when making a choice about CRC screening
- Tip Sheet: Applying for Health Insurance: a step-by-step process for applying for health insurance using the Health Insurance Marketplace
- How to Find a Primary Care Provider: tips for steps to take to find a PCP
- Financial Assistance Options: contact information for various sources of financial assistance for CRC screening
- Letter for Insurance Company: a letter for participants to share with their insurance company to help with coverage for CRC screening

The SCP will be personalized for each participant: the study team will generate a draft SCP using previously collected data from the CCSS, an advanced practice registered nurse (APRN) will review the SCP for errors, and the study manager will upload the SCP to the website.

#### Text-message intervention

**Fig. 2:**
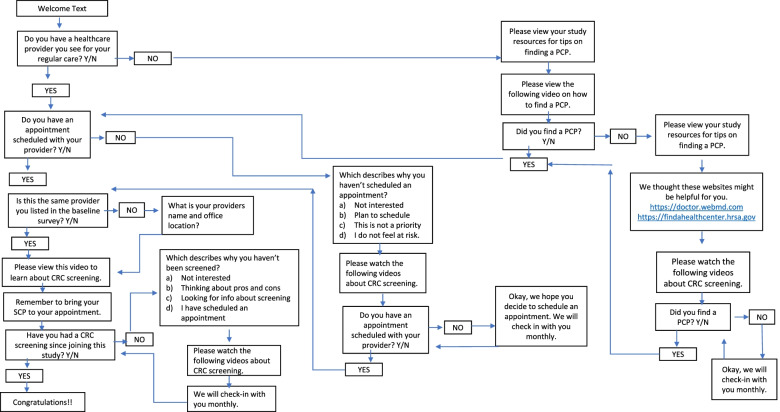
Text message content overview

Based on feedback from our PCP advisory board, comprised of 9 General Internists, Family Physicians and Gynecologists, and preliminary data from the EMPOWER study [32] which utilized a similar text-message intervention to encourage breast cancer screening, we designed a interactive text-message intervention via the Mosio texting platform (Fig. 2). The text-message intervention employs the Patient Activation Model, which suggests that activated patients are better prepared to participate in self-management [18].

The texts include questions to be answered by the participants, educational links, reminders to view the study resources via the CCSS myLTFU website, and links to four videos on important topics. The following videos ranged from 60 to 90 seconds in length and were scripted and filmed by the study team using Zoom: 1) The Importance of Regular PCP Appointments for Cancer Survivors, 2) Perspective from a Cancer Survivor, and 3) Expected Cost of a Colonoscopy or multitarget stool DNA test.

In addition, in collaboration with the video animation company Splainers, we created a 60-second video explaining the key attributes of both a colonoscopy and a multitarget stool DNA test. Based on how they answer the questions, participants will receive study texts for a duration of 2–11 months. The text message intervention was thoroughly tested and reviewed by the study team to ensure accuracy.

#### Primary care provider resources

Informed by PCP activation strategies used by other studies with a similar focus to increase cancer screening rates [13, 14], the study team created the following primary care provider resources:

- Cover Letter: summary of patient’s SCP, contact information for study team
- Executive Summary: overview of current CRC screening recommendations
- FAQ Page: answers to common questions such as “How often should high-risk patients be screened for colorectal cancer?”
- Insurance Letter Template: a letter to share with a patient’s insurance company to help with coverage for CRC screening

The PCP resources will be faxed along with the baseline survey.

### Outcome measures

Table 2 provides an overview of effectiveness and implementation outcomes, including operational definitions, data sources, and data collection timing details.

**Table 2:**
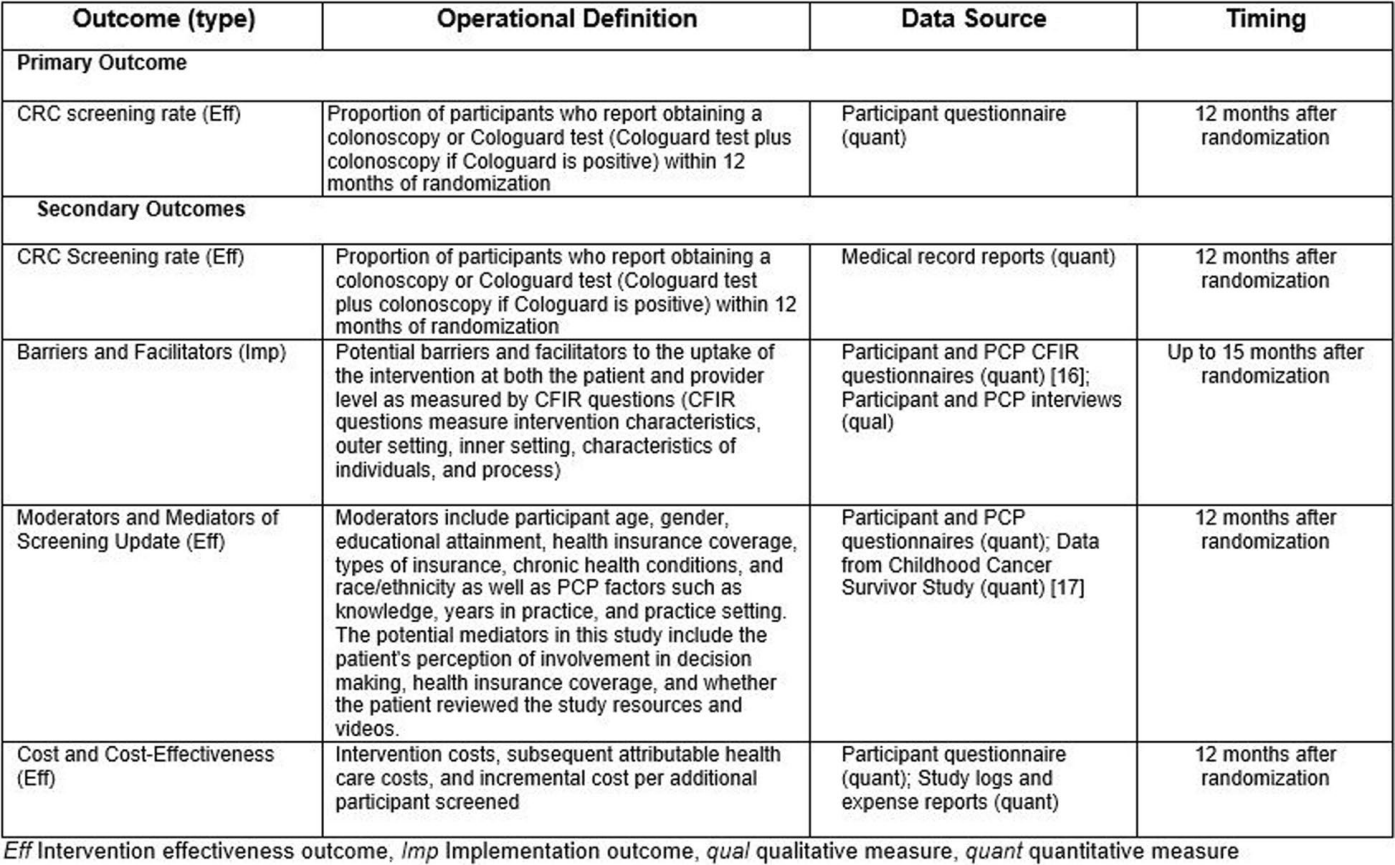
Primary and Secondary outcome measures

#### Effectiveness outcomes

##### CRC screening rate

The primary effectiveness outcome is CRC screening completion within 12 months after randomization. CRC screening completion is defined as the proportion of participants who self-report obtaining a colonoscopy or multitarget stool DNA test (multitarget stool DNA test plus colonoscopy if multitarget stool DNA test is positive). A secondary effectiveness outcome is CRC screening completion by 12 months after randomization, as measured through medical record report confirmation. Study team members will reach out to retrieve a medical record report to confirm CRC screening for each participant who reports that screening was completed within 12 months of randomization.

Through participant questionnaire items, we will assess key factors which may be associated with CRC screening completion (Table 3). Participant activation will be measured through the Patient Activation Measure (PAM) short form [33]. PCP activation will be evaluated through targeted questions in the 12-month PCP questionnaire. Intention to have CRC screening will be measured through questionnaire items developed based on the Theory of Planned Behavior [34]. In order to evaluate the benefits and barriers to CRC screening and the perceived seriousness of susceptibility to CRC, we adapted questions from the Champion Benefits Scale for Mammography Screening [35]. We will use the Patient-Reported Outcomes Measurement Information System (PROMIS) questionnaire to measure self-efficacy [36, 37].

**Table 3:**
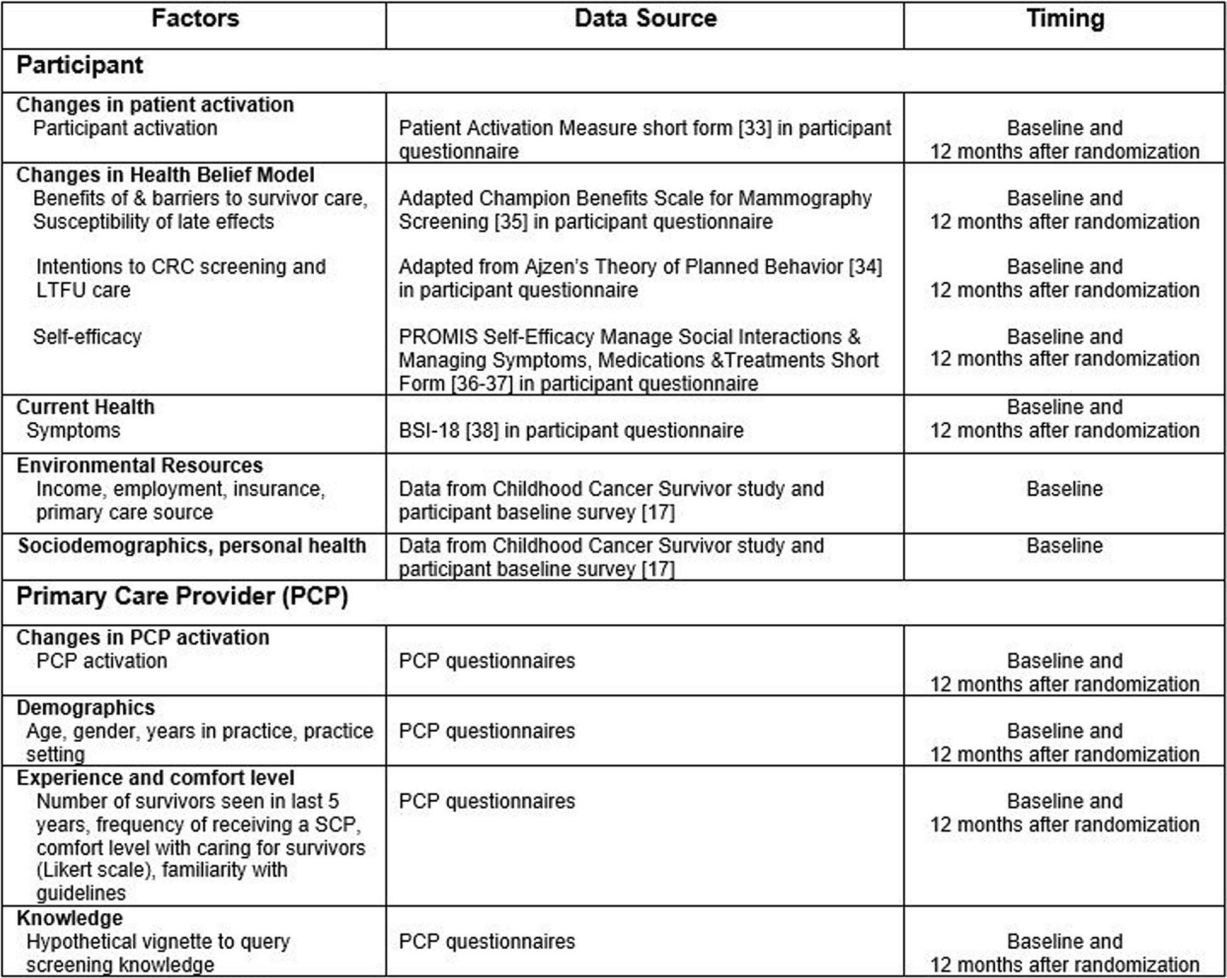
Key factors associated with CRC screening completion

Other participant-level factors that will be measured via targeted questions in the baseline and 12-month questionnaires include current health, environmental resources, sociodemographics, and personal health. Current psychological health will be measured through the Brief Symptom Inventory (BSI-18) [38]. Other PCP-level factors that will be evaluated include demographics, experience and comfort level providing care to cancer survivors, and knowledge about the healthcare needs of childhood cancer survivors.

##### Moderators and mediators

Another secondary effectiveness outcome is the assessment of potential moderators and mediators [39]. In this study, the potential moderators include patient characteristics, such as age, gender, educational attainment, health insurance coverage, types of insurance, chronic health conditions, and race/ethnicity as well as PCP factors such as knowledge, years in practice, and practice setting. The potential mediators may include the patient’s perception of involvement in decision-making, health insurance coverage, and whether the patient reviewed the study resources and videos. We will measure these moderators and mediators via the patient questionnaires, PCP questionnaires, and previously collected data from the CCSS.

##### Cost and cost effectiveness

The final secondary effectiveness outcomes are the cost and cost-effectiveness of the intervention; these will be measured by estimating intervention costs and the costs of subseuqnt health services attributable to the intervention. Costs for each component of the intervention (e.g. materials, text messaging design, etc.) will be collected to estimate the cost per person. These costs will be tracked by the study team via study logs and expense reports. In the 12-month survey, we will ask participants about routine or acute medical visits, whether any visit was for gastrointestinal symptoms or conditions or concern about CRC risk, and whether such visits occurred before or after screening, among those who report colonoscopy or multitarget stool DNA test. This data will allow us to quantify the costs of health care services whose use may be attributable to the intervention. Incremental cost-effectiveness ratios will be estimated as the incremental cost per additional participant screened by 12 months, comparing the three study arms.

#### Implementation outcome

We will employ a mixed methods approach to assessing the implementation process from both the participant and the provider perspective.

##### Barriers and facilitators

The secondary implementation outcome is the assessment of barriers and facilitators to the uptake of the intervention. The CFIR provides a structure for evaluating an implementation process [16]. We selected constructs from each of the 5 major CFIR domains (intervention characteristics, outer setting, inner setting, characteristics of individuals and process) as they relate to our intervention (Table 4). PCPs will be asked CFIR questions as part of the 12-month questionnaire and as a component of the end of study interview. In order to develop a more comprehensive understanding of the barriers and facilitators to the implementation of the intervention, we have also included CFIR questions in the 12-month questionnaires and interviews for the participants.

**Table 4:**
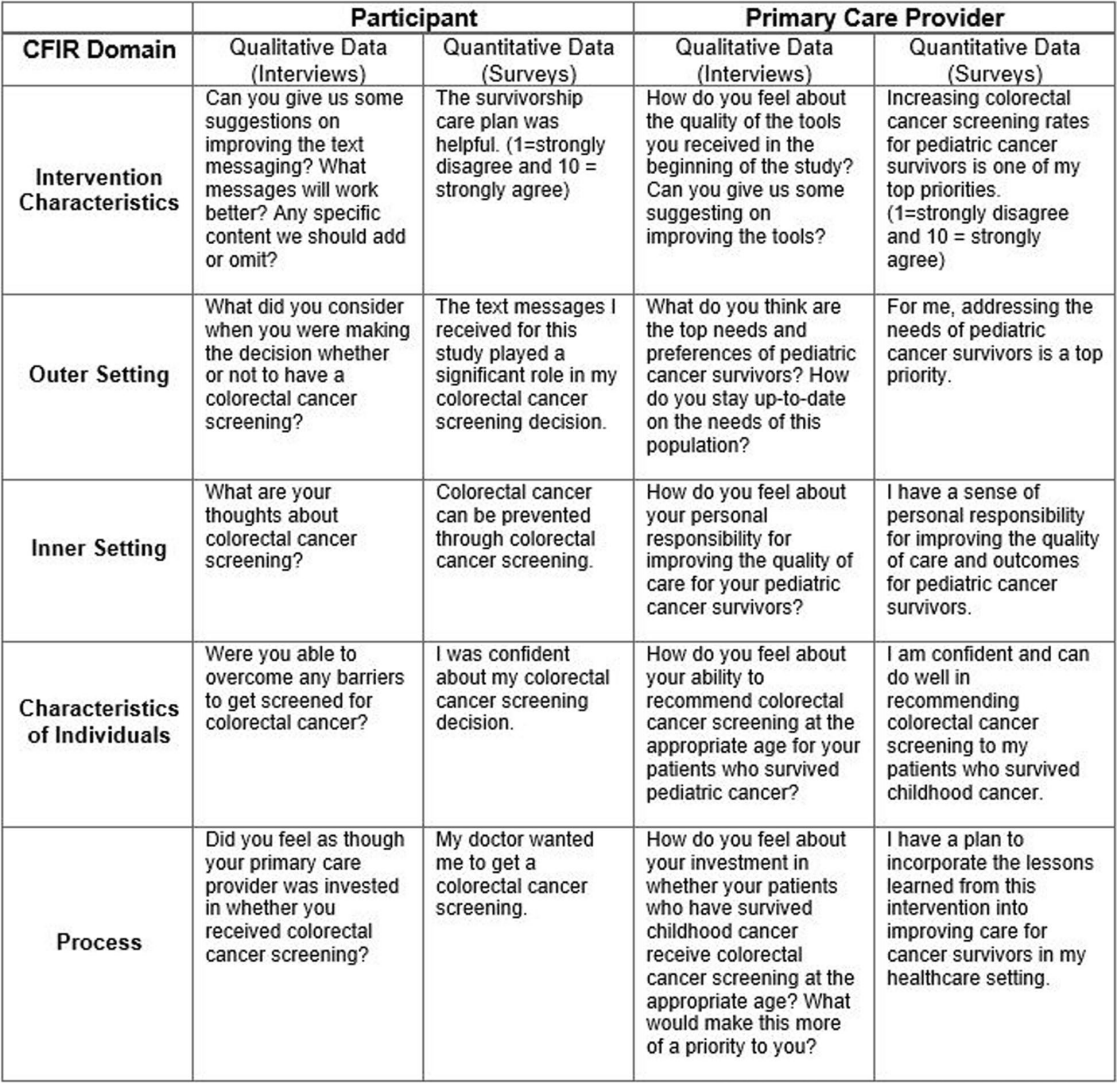
CFIR items for participants and primary care providers

A sample of 20 participants in each intervention arm will be selected for the CFIR interviews. Interviewees will be selected based on their CRC screening behavior since enrolling on the study. In each intervention arm,10 participants who completed CRC screening and 10 participants who did not complete CRC screening will be interviewed. Similarly, a sample of 20 PCPs from each intervention arm will be selected for a CFIR interview. In each intervention arm, 10 PCPs of participants who completed CRC screening since joining the study, and 10 PCPs of participants who did not complete CRC screening since joining the study will be interviewed. Interviews will be conducted and recorded via Zoom, and the study team will transcribe all interviews using Temi, a speech transcription service available online.

### Data sources

We will use a REDCap database, DatStat database, questionnaires, interviews, medical records, previously collected CCSS data, and study logs to collect data for the ASPIRES study. At the conclusion of the study, the statistics team at Memorial Sloan Kettering will have access to the final trial dataset.

#### REDCap database

Personnel at the University of Chicago will manage a secure REDCap database which will contain PCP and medical office staff questionnaire data, medical record confirmation results, all interview transcripts, and select participant questionnaire data. The PROMIS [36, 37], PAM [33], and BSI-18 [38] surveys are components of the participant baseline and 12-month questionnaires; these surveys will be scored, and the scores will be stored in the REDCap database.

#### DatStat database

St. Jude personnel will maintain the DatStat database which will store all participant baseline and 12-month questionnaire data. The University of Chicago team members will have access to this data to score select surveys (see REDCap database above).

#### Questionnaires

We will collect baseline and 12-month participant questionnaires, baseline and 12-month PCP questionnaires, and a 12-month medical office staff questionnaire. To develop the participant questionnaires, the study team compiled all items in existing instruments such as the PAM [33], PROMIS [36, 37], and BSI-18 [38]. The study team also included modified items from a mammography screening tool [35], items developed based on Aizen’s Theory of Planned Behavior [34], CFIR items developed with the input of an expert on the study PCP advisory board [16], and other items designed to capture all significant variables such as CRC screening completion, moderator and mediator data, and cost information.

To create the primary care provider questionnaires, the study team modified an 18-item survey which was previously administered to measure family physician knowledge regarding the care of childhood cancer survivors [14]. The study team edited this questionnaire to include CFIR items and questions to measure key demographic variables [16]. The medical office staff questionnaire was designed by the study team to gather additional feedback on the implementation of the intervention from the helpful perspective of a staff member working alongside the PCPs. All questionnaires were reviewed by the study PCP advisory board which includes many clinician-researchers, two psychologists, multiple primary care providers, a research nurse with expertise in CFIR, a cancer survivor/social worker, among other experts.

#### Interviews

We will conduct structured interviews with 20 primary care providers, 20 medical office staff, and up to 20 participants from each intervention arm to collect data on the implementation process (see Implementation Outcome section above for more details). We used the CFIR to guide the creation of the interview guides [16]. All interviews will be transcribed using Temi, and the transcription will be uploaded into the REDCap database.

#### Medical record reports

CRC screening reports for participants who note that they had a colonoscopy and/or multitarget stool DNA test will be obtained by contacting the ordering provider. In the 12-month questionnaire, participants will be asked to provide contact information for the healthcare provider who ordered their CRC screening. Study personnel will obtain the CRC screening reports and input the data into the REDCap database.

#### CCSS data

Age, gender, diagnosis of chronic health conditions, and race/ethnicity data for enrolled participants will be provided from previously collected data by the CCSS [17]. Since all ASPIRES study participants were previously enrolled on the CCSS, we have access to data from the surveys administered as part of this study. The CCSS Statistics and Data Center at Fred Hutchinson will send this data via a spreadsheet to the ASPIRES study statistical team at Memorial Sloan Kettering Cancer Center.

#### Study logs

Study personnel will track the cost of intervention components (e.g., material creation, text message design, personnel time to accomplish key tasks etc.) via a study log spreadsheet.

### Data analysis plan

#### Primary efficacy analysis

The primary analysis involves comparing the proportion of participants who report obtaining a colonoscopy or negative multitarget stool DNA test report or positive multitarget stool DNA test report in combination with a confirmed colonoscopy (completed screening test) in each intervention arm to the control arm. To account for the randomization strata and the sampling scheme, this difference will be estimated by taking a weighted average of the differences in stratum-specific proportions. The test of the difference in proportions will be done using a Cochrane-Mantel-Haenszel test [40].

#### CFIR analysis

For the CFIR quantitative data, we will calculate a mean score of each statement and compare the arm differences using ANOVA (Analysis of Variance) [16]. For the CFIR qualitative data, we will use a template analysis approach to code and organize the data. Template analysis is a form of thematic analysis that emphasizes the use of hierarchical coding but balances a relatively high degree of structure in the process of analyzing text data with the flexibility to adapt it to the needs of a particular study [41]. We will develop a codebook, which will include CFIR constructs. The codebook will provide the operational definition of each code, and the inclusion and exclusion criteria. The coder will interpret the data first and then apply the CFIR code that reflects a potential barrier or facilitator being described. We will have four different coders who have experience in qualitative research to code the same data. In addition, we will evaluate the interrater reliability. The four coders will meet to discuss and reach a consensus on any coding discrepancies. Finally, using a convergent mixed-methods approach [42], we will merge the quantitative and qualitative data, organized by the 5 CFIR domains to evaluate which constructs were associated with, or more prevalent among patients who had colonoscopy or multitarget stool DNA test and those who did not (test yes v. test no), those who had positive multitarget stool DNA test and had a colonoscopy and those who did not complete a colonoscopy, and those whose PCPs ordered a test and those who did not (regardless of whether a survivor had a test); this will allow us to comprehensively evaluate facilitators and barriers to ordering and completing CRC screening to make recommendations for future adaptation and sustainability.

#### *Moderators and mediators* analysis

Since our outcome variable is dichotomous (screened or not screened), we will use logistic regression to assess the moderator effect of these characteristics on the relationship between the intervention and the primary outcome: log [Pi (1 – Pi)] = β0 + β1X + β2Z + β3 XZ + e, where Pi is the probability for patient i, β1 is the coefficient of the predictor X, β2 is the coefficient of the moderator Z, β3 is the coefficient of the product term XZ, and e is the error. We will use the likelihood ratio test to compare the nested models (the first-order model log [Pi (1 – Pi)] = β0 + β1X + β2Z + e and the higher-order model log [Pi (1 – Pi)] = β0 + β1X + β2Z + β3 XZ + e). The change in the − 2 Log Likelihood (−2LL) will be examined to see whether adding the product term to an existing additive regression equation would significantly increase the predictability. When the change is significant, a moderator effect is considered.

To explore the mediator effect, we will follow MacKinnon et al’s [43] recommendations and examine potential factors in a post hoc manner. We will use fit path analysis models using the statistical packages LISREL or EQS, inspect path coefficients, and choose the appropriate statistical tests of mediating effects according to MacKinnon et al. [43]. We may also consider testing model equivalence to examine whether the mediating path model is equivalent across the control and intervention arms. While several different tests have been used to test mediating factors, MacKinnon et al. [43] recommend using tests developed from the product of coefficients methods, where the parameters are estimated using regression, and the standard error of their product is obtained by the delta method. This test can be readily implemented using software made available by MacKinnon et al. [43]. All analyses will be adjusted for the stratification factors used at randomization.

#### Cost and cost-effectiveness analysis

In addition to estimating the costs of replicating the intervention, we will also perform a limited cost-effectiveness analysis. We will estimate the cost per additional screening completed, comparing the 3 study arms. Consistent with accepted methods of cost-effectiveness analysis, the numerator of each incremental cost-effectiveness ratio will include both intervention costs, and the costs of health care services, other than the recommended CRC screening, whose use may be attributable to the intervention. This component – attributable health care services – will be estimated by multiplying reported health care visits (measured in the 12-month participant survey) by unit cost estimates from the Medicare Physician Fee schedule. Although most study participants will not be Medicare beneficiaries, Medicare’s reimbursement methodology was developed to reflect true resource cost, thereby serving as a proxy for unit cost [44]. In sensitivity analysis, we will evaluate a range of unit cost estimates, both lower and higher than the average Medicare reimbursement level. Patient time and travel costs for medical visits will be estimated from the literature [45].

Because the measure of effectiveness in this analysis is additional screening completed, the cost-effectiveness analysis will not include the cost of screening or subsequent costs associated with CRC diagnosis and treatment. Resource utilization and cost data are typically skewed, and therefore the sample size of the trial will likely be insufficient to detect significant differences in costs between study arms [19]. Sensitivity analysis will be used to assess the impact of assumptions and uncertainty on results and conclusions [46, 47]. This analytic approach is appropriate in economic studies that “piggyback” randomized trials [48].

### Sample size and power

The primary aim involves two pair-wise comparisons of equal importance. We fix the probability of a Type I error at 0.025 for each comparison in order to maintain the overall probability of a Type I error at 0.05. We powered the study to detect a difference of 20% for each pair-wise comparison, assuming equal randomization to each of the three arms, and that approximately 20% of participants in the control arm will be adherent with recommended CRC screening. Table 5 shows the power we expect to have using two-sided Cochrane-Mantel-Haenszel tests and indicates that we should have sufficient power to detect a clinically meaningful difference even if the proportion of participants in the control arm with the primary endpoint is somewhat different than we expect.

**Table 5:**
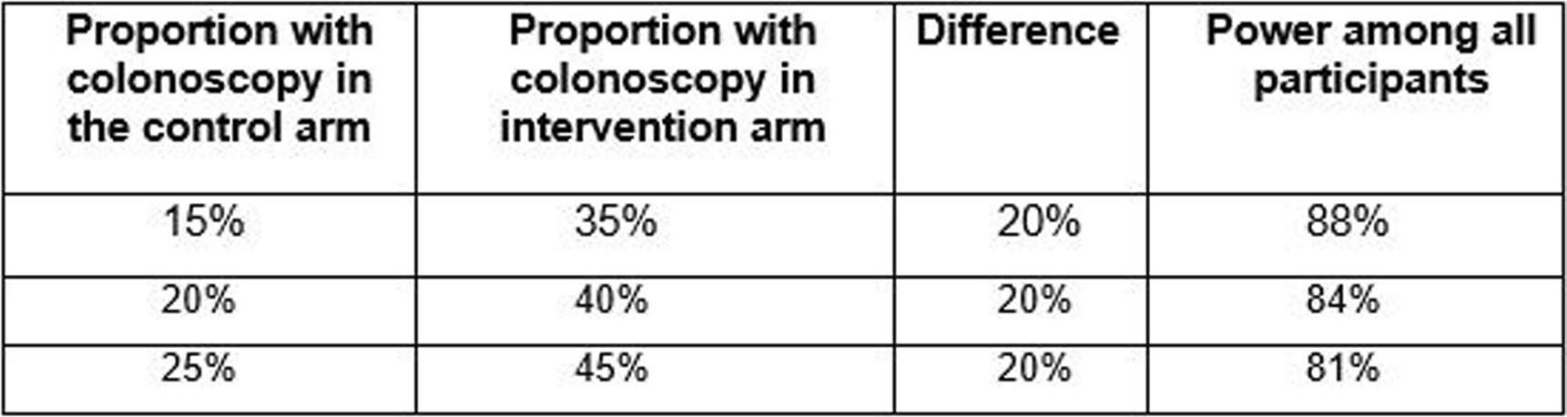
Power to detect a difference between intervention and control arms

## Discussion

We describe the first Type II Hybrid Implementation Study to our knowledge to test the efficacy of a mHealth intervention to improve CRC screening in childhood cancer survivors at high risk for CRC due to previous exposure to radiation to the abdomen, pelvis, spine, or TBI. The importance of this study cannot be understated in that cancer survivors with this radiation exposure are almost four times more likely to develop CRC, a preventable cancer, compared to the general population [6].

### Limitations and anticipated challenges

There are several limitations to this protocol. The burden to understand the effectiveness of the mHealth intervention requires robust participation among the PCPs of enrolled patients and their interest in reviewing the study materials and completing the questionnaires and interviews. While PCPs generally value research and consider it an important pathway towards the generation of useful clinical knowledge, they also find it to be administratively burdensome [49]. Based on feedback from our PCP Advisory Board on how to best address this potential issue, we have ensured that all PCP study materials are concise, have included a gift card for participation in each study component, and have developed a structured follow-up plan to collect any missing data.

We also recognize that apprehension about the cost of CRC screening may impact the willingness of participants to follow through with their screening. Previous studies indicate that concern about cost is a leading patient-reported barrier to CRC screening [50, 51]. Since many insurance companies are unaware that people at high-risk for developing CRC should begin screening when they are 30 years old, it is possible that younger participants could have a high out-of-pocket cost for this screening. To address this challenge, we provide all participants with a templated insurance letter they can use to communicate why CRC screening should be covered by their insurance company. As part of their study materials, we also supply participants with a resource which lists organizations that provide financial assistance for CRC screening. In addition, participants in the intervention arms receive a video about CRC screening cost as part of the text-message intervention.

### Potential for impact and implications

There is a critical need to increase the rate of CRC screening among high-risk cancer survivors. This study will advance our understanding of CRC prevention within the high-risk childhood cancer survivor population by evaluating both the effectiveness of an mHealth intervention and assessing implementation strategies that facilitate or impede intervention outcomes. The study design -a three-arm, hybrid type II effectiveness and implementation trial- will inform the development of specific implementation strategies for the post scale-up of this study and will provide generalizable, comprehensive, and clinically useful data for decision makers to ensure future sustainability.

## Supplementary Information

**Additional file 1.** This file includes a sample informed consent form

**Additional file 2.** This file includes a sample of each participant resource that is provided to study participants

## Data Availability

Not applicable.

## Abbreviations

ANOVA: Analysis of variance
APRN: Advanced Practice Registered Nurse
ASPIRES study: Activating Cancer Survivors and their Primary Care Providers to Increase Colorectal Cancer Screening Study
BSI: Brief symptom inventory
C: Control
CCSS: Childhood Cancer Survivor Study
CFIR: Consolidated Framework for Implementation Research
COG: Children’s Oncology Group
CRC: Colorectal cancer
mHealth: Mobile health
PA: Patient activation
PAM: Patient Activation Measure
PCP: Primary care provider
PROMIS: Patient-Reported Outcomes Measurement Information System
RT: Radiation Therapy
SIR: Standardized Incidence Ratio
SMN: Subsequent malignant neoplasms
SPN: Subsequent Primary Neoplasm
SCP: Survivorship Care Plan
TBI: Total Body Irradiation
