# Supplementary material for: Implementing a mHealth intervention to increase colorectal cancer screening among high-risk cancer survivors treated with radiotherapy in the Childhood Cancer Survivor Study (CCSS)
Source: BMC Health Serv Res. 2022 May 23;22:691. doi: 10.1186/s12913-022-08082-3 (PMC9128150; doi:10.1186/s12913-022-08082-3)
Supplement: Supplementary file 1 — Additional file 1. This file includes a sample informed consent form [file 12913_2022_8082_MOESM1_ESM.pdf]

The UNIVERSITY OF CHICAGO
The Division of the Biological Sciences • The University of Chicago Medical Center

CONSENT FOR PARTICIPATION IN A RESEARCH PROTOCOL

STUDY TITLE: Activating Cancer Survivors and their Primary Care Providers to Increase Colorectal Cancer Screening (ASPIRES) Study

Doctors Directing Research: Tara Henderson, MD, MPH (University of Chicago), Karen Kim, MD, MS (University of Chicago), and Greg Armstrong, MD (St. Jude Children's Research Hospital)
Address: University of Chicago (5841. S. Maryland Ave. 57th Street, MC 4060, Chicago, IL 60637), St. Jude Children's Research Hospital (262 Danny Thomas Place, Memphis, TN 38105)
Telephone Number: University of Chicago (773) 702-6808; St. Jude Children's Research Center (800) 775-2167
Email: ASPIRESStudy@bsd.uchicago.edu

KEY INFORMATION

As a participant in the Long-Term Follow-Up (LTFU) Study being conducted by St. Jude Children's Research Hospital, you are invited to participate in a research study called the ASPIRES study. This study is being funded by the National Cancer Institute. We are asking you to choose whether or not to volunteer for a research study about colorectal cancer (CRC) screening among cancer survivors. The purpose of this section is to give you key information to help you decide whether to participate. We have included detailed information after this section. You can ask the research team questions before you choose to participate. If you have questions later, the contact information for the research investigator in charge of the study is above and you can feel free to email the study team at ASPIRESStudy@bsd.uchicago.edu.

WHAT IS THE STUDY ABOUT AND HOW LONG WILL IT LAST?

By doing this study, we hope to learn how to encourage cancer survivors to get screened for colorectal cancer at the recommended age. Your participation in this research will last up to fifteen months and will include the completion of surveys and possibly completing a short interview. Your standard of care will not be altered in any way.

WHAT ARE KEY REASONS YOU MIGHT CHOOSE TO VOLUNTEER FOR THIS STUDY?

The main benefit from participating in this study is that you will learn more about why colorectal cancer screening is important for your health. For a complete description of benefits, refer to the Detailed Consent listed below.

WHAT ARE KEY REASONS YOU MIGHT CHOOSE NOT TO VOLUNTEER FOR THIS STUDY?

The main risk to you in taking part in this study is the loss of confidentiality. Study records that identify you will be kept confidential. Electronic files will reside on secure, HIPAA-compliant servers in password
protected data sets. For a complete description of risks, refer to the Detailed Consent. Also, please note that no individuals will be identified in study reports.

DO YOU HAVE TO TAKE PART IN THE STUDY?
Taking part in this study is voluntary. If you decide to take part in the study, it should be because you really want to volunteer. You will not lose any services, benefits or rights you would normally have if you choose not to volunteer.

You may choose not to participate at any time during the study. You will not lose any services, benefits or rights you would normally have if you choose to leave the study. If you do not sign this form, you will not receive the research-related intervention(s).

WHAT IF YOU HAVE QUESTIONS, SUGGESTIONS OR CONCERNS?
The person in charge of the study is Dr. Tara Henderson of the University of Chicago. If you have questions, suggestions, or concerns regarding this study or you want to withdraw from the study her contact information is: (773) 702-6808.

For questions about your rights as a research subject, please contact the University of Chicago BSD IRB at 773-702-6505.

DETAILED CONSENT

WHAT IS INVOLVED IN THE STUDY?
About 945 people throughout the United States and Canada will take part in this study.

If you agree to participate in this study, you will complete questionnaires in the beginning of the study and again 12 months after you enroll on the study. The questionnaires will be accessed via an online portal, and they will take about an hour to complete at both baseline and at 12 months after you enroll in the study. The questionnaires cover topics such as your employment, current health, health care access, and cancer screening practices. The 12-month questionnaire will also include the option to upload your Explanation of Benefits document from your health insurance plan. Your standard of care will not be altered in any way.

If you consent to be part of this study, your doctor will be asked to provide information about your care.
Intervention
You will be randomly assigned to one of three groups. Randomization means that the group you are in is based on chance. It is like flipping a coin, except that it is done by a computer. You and your primary care doctor will not get to pick which group you are in. See below for more details about the study procedures for each group.

Group 1:
- You will receive electronic colorectal cancer prevention and screening materials.

Group 2:
- You will receive electronic colorectal cancer prevention and screening materials.
- You will receive text messages with educational links and videos
- You may be contacted to complete a short, phone-based interview. This interview may be recorded so that it can be transcribed for data analysis. After the recordings are transcribed and data analysis is complete, the recordings will be destroyed.

Group 3:
- You will receive electronic colorectal cancer prevention and screening materials.
- You will receive text messages with educational videos
- Your primary care provider will be faxed screening recommendations for you.
- You may be contacted to complete a short phone-based interview. This interview may be recorded so that it can be transcribed for data analysis. After the recordings are transcribed and data analysis is complete, the recordings will be destroyed.

This is an outlined summary of the ASPIRES Study:

**Timeline**	**Component**
Study Entry	• Baseline Questionnaire<br>• Random assignment to Group 1, Group 2, or Group 3
After Study Entry	• Electronic cancer prevention materials<br>• Text messages (depending on group assignment)

12 Months After Study Entry	• End of study Questionnaire
Up to Fifteen Months After Study Entry	• Some participants are interviewed

In the future, identifiers associated with your data could be removed from the data. The de-identified data could then be used for future research by our research team or other researchers without notifying you or asking your permission for this use.

The results from this study will not be shared with you.

Dr. Henderson may decide to take you off of the study without your consent if:

- You are unable to meet the requirements of the study or your medical condition changes;
- New information becomes available that indicates that participation in this study is not in your best interest; or
- If the study is stopped.

WHAT ARE THE RISKS OF THE STUDY?
Some participants may find the questionnaires to be boring, tiring or frustrating. You will be asked questions about things that might be upsetting or may remind you about other problems. Extreme distress is unlikely, but if it does occur, referrals for counseling can be made.

Also, there is a risk of loss of confidentiality. Please see below under "What about Confidentiality?" for more information.

There may be other risks that could arise which are not reasonably foreseeable. If new information becomes available which could influence your willingness to continue, this new information will be discussed with you.

ARE THERE ANY BENEFITS TO TAKING PART IN THE STUDY?
This study will provide you with information about recommended medical screening. We hope that your participation in the study may benefit other people in the future by helping us learn more about the most effective intervention to encourage cancer survivors to get screened for colorectal cancer.

WHAT OTHER OPTIONS ARE THERE?
You may choose not to participate in this study. The decision whether or not you wish to participate in this study will not affect your care or continued participation in the LTFU Study.

WHAT ARE THE COSTS?
Clinical services provided during a clinical research study are either research-related or considered part of usual medical care for patients with your disease or condition. Tests, procedures, and activities that are ordered by your medical care team to monitor your disease or condition (whether you are participating in a clinical trial or not) are described as 'usual medical care'. 'Research-related' is the term used to describe any tests, procedures, or activities (interviews, surveys) that you are being asked to undergo only because of your participation in this clinical research study.

You or your insurance will be financially responsible for the costs of your usual, ongoing medical care. This often includes regular visits with your doctor, lab tests, and other tests and procedures deemed

medically necessary by your care team, such as a screening colonoscopy exam. Financial responsibilities for routine care may include deductibles and co-payments and this care will be subject to all the same requirements and restrictions of your insurance.

There will be no additional costs to you or your insurance company resulting from your participation in this research study. However, you or your insurance company will be responsible for costs related to your usual medical care which includes the recommended colorectal cancer screening test (s).

WILL I BE PAID FOR MY PARTICIPATION?
For completion of the study questionnaires in the beginning of the study, you will receive a $50 Amazon gift card. Upon completion of the questionnaires 12 months after you consent to the study, you will receive another $50 Amazon gift card. If you are selected for a follow-up interview after the completion of the clinical trial, you will receive an additional $50 Amazon gift card.

WHAT ABOUT CONFIDENTIALITY?
There is a risk of potential loss of confidentiality. To minimize this risk, study records that identify you will be kept confidential. Data will be stored in locked files with limited access to selected authorized staff. Electronic files will reside on secure, HIPAA-compliant servers in password protected data sets and procedures for all transfer of electronic data will meet current security standards. If information about this study is published or presented at scientific meetings, your name and other personal information will not be used.

We may share coded study data with collaborators at St. Jude Children's Research Hospital, Memorial Sloan Kettering Cancer Center, and other LTFU collaborating researchers. This information is being sent for data analysis purposes. Please note that at the end of the study you will be asked to sign a separate authorization document to authorize the study team to access your colonoscopy or Cologuard medical records. Additionally, if we use or disclose any protected health information, you will be asked to sign a separate authorization document.

Your records may be reviewed by federal agencies whose responsibility is to protect human subjects in research including the Office of Human Research Protections (OHRP). Representatives of the University of Chicago, including the Institutional Review Board (a committee that oversees the research) and the Office of Clinical Research may also view the records of the research.

. The study results will be kept in your research record and be used by the research team until completion of the study.

Data from this study may be used in medical publications or presentations. Your name and any other identifying information will be removed before this data is used. If we wish to use identifying information in publications, we will ask for your approval at that time. We may also share de-identified data with collaborators or others for research purposes.

WHAT ARE MY RIGHTS AS A PARTICIPANT?

You will receive an emailed copy of this consent form.

CONSENT
The experimental procedures have been identified and no guarantee has been given about the possible results. You can access and download a copy of this consent from the ASPIRES Study portal.

I agree to participate in this study. My participation is voluntary and I do not have to agree if I do not want to be part of this research study.

Clicking the button below indicates that you agree to be in this study.

AGREE TO PARTICIPATE NO THANKS
