# Supplementary material for: Implementing a mHealth intervention to increase colorectal cancer screening among high-risk cancer survivors treated with radiotherapy in the Childhood Cancer Survivor Study (CCSS)
Source: BMC Health Serv Res. 2022 May 23;22:691. doi: 10.1186/s12913-022-08082-3 (PMC9128150; doi:10.1186/s12913-022-08082-3)
Supplement: Supplementary file 2 — Additional file 2. This file includes a sample of each participant resource that is provided to study participants [file 12913_2022_8082_MOESM2_ESM.pdf]

ASPIRES

Survivorship Care Plan Date:

**Name:**	**CCSSID#**
**Date of Birth:**	**Age of Diagnosis:**
**Cancer Diagnosis:**	
**Treatment Center:**	
**Cancer Treatment**	
**Radiation dates:**	
**Radiation locations and doses:**	
**Chemotherapy drugs and doses:**	
**Screening Recommendations***	

If you note any errors in this document or have questions, please contact

Dr. ________________ at __________________________ at ______________________
or
Dr. Tara Henderson or Casey DeBias, APN at ASPIRES Study/University of Chicago at (773) 702-6808, ASPIRESStudy@bsd.uchicago.edu.

*For information about other health risks associated with childhood cancer and health screening recommendations, see the CureSearch Children's Oncology Group Long-Term Follow-Up Guidelines at http://www.survivorshipguidelines.org.

ASPIRES

How to use your Survivorship Care Plan (SCP)

See below for four recommended steps to take as you view your SCP.

1

Download and save

Download and save your Survivorship Care Plan (SCP) to your personal device so you can reference it as needed. Don't worry, your SCP will also be available in your ASPIRES portal if needed.

2

Review content

Review the treatment information in your SCP and learn about your screening recommendations. Please contact us if you have any questions about your SCP.

3

Provide your primary care provider with a copy

Bring your SCP with you to any appointments with your primary care provider (the healthcare provider you see for your regular care) to discuss your treatment history and screening recommendations. You can also ask to have a copy put in your medical records.

4

Learn more

Learn more about recommendations for survivors by viewing the Children's Oncology Group Long-Term Follow-up Guidelines at http://www.survivorshipguidelines.org. Share this link with your primary care provider too!

Potential Benefits and Other Considerations of Colorectal Cancer Screening

Among people who have been treated with total body irradiation and/or radiation to the pelvis, abdomen or spine

- People who were treated with body irradiation and/or radiation to the pelvis, abdomen or spine are almost four times more likely to develop colorectal cancer (CRC) compared to the general population with the elevated risk evident by the age of 30

- Almost 80% of cancer survivors at elevated risk for colorectal cancer are not screened according to the current recommendations

- Subsequent malignant neoplasms (SMN) are the leading cause of premature death in cancer survivors.

- Through screening, colorectal cancer can be prevented. When detected at an early stage through a screening test, colorectal cancer is highly curable.

The **Children's Oncology Group** (national experts in survivor health issues) recommends that people with your radiation history have a colorectal cancer screening test starting 5 years after radiation or at age 30 (whichever occurs last); you should have a colonoscopy every 5 years, or a cologuard test every 3 years. There are known potential benefits and other considerations of these tests. It is important that you are aware of these points in order to make an informed decision.

POTENTIAL BENEFITS

- A colonoscopy or cologuard test can detect polyps before they become cancer and can detect colorectal cancer at an early stage.

- A colonoscopy or cologuard test should be covered by your health insurance.

OTHER FACTORS TO CONSIDER

- People screened for colorectal cancer may feel anxious to have these tests and wait for the results.

- Some people may experience discomfort during a colonoscopy, but this test is normally not painful.

- If you decide to have a colonoscopy, your doctor will discuss the preparation needed for this test (this normally involves drinking a liquid that causes diarrhea which helps to rid the colon of its contents, following a special diet the day before the exam, and adjusting your medications for at least a week before the exam).

- Since you are given medication to help you stay relaxed (this is called sedation) during a colonoscopy, you will need someone to give you a ride home after this screening test.

- Cologuard is a screening test that is done at home and does not require any preparation. However, if you have an abnormal test, you need to have a colonoscopy.

ASPIRES

TIP SHEET: APPLYING FOR HEATH INSURANCE

If you don't have health insurance through your job, your spouse's job, a government plan, or the VA, please see below for some steps to follow to apply for health insurance.

1) If you live in the United States, are a U.S. citizen or national, and are not incarcerated, you can apply for health insurance through the Health Insurance Marketplace.

   Note: people with varying immigration statuses quality to use the Marketplace. For a detailed list of qualifying immigration statuses, please see https://www.healthcare.gov/immigrants/immigration-status/.

2) To access the Health Insurance Marketplace, go to www.HealthCare.gov.

   Depending on where you live, you can apply for benefits through the ACA Health Insurance Marketplace or you will be directed to your state's health insurance marketplace website. Marketplaces, prices, subsidies, programs, and plans vary by state.

   You can also contact the Marketplace Call Center at 1-800-318-2596 or TTY at 1-855-889-4325.

3) Next, you can create a Marketplace account, and submit your application. You will receive "eligibility results" which will provide you with information about qualifying healthcare plans.

4) Then, you can compare health plans/prices, and enroll in the plan that best fits your needs.

5) After you enroll, you will receive a mailed packet which includes your insurance card and details about your health plan.

Resources:
Many resources (including a quick start guide and a health insurance calculator) to help you through this process are available at www.HealthCare.gov.

ASPIRES

HOW TO FIND A PRIMARY CARE PROVIDER

Primary care physicians usually see their patients on a regular basis and look for symptoms that a patient may not notice. Developing a relationship with your primary care physician can be very helpful to you. If you do not already have a primary care physician, here are some helpful hints on finding one.

How to find and choose a primary care physician:

- Check which primary care physicians are local to you and in your insurance provider network. Your health insurance plan may keep an online list of network primary care physicians who are accepting new patients.

- People often ask their friends, families, and coworkers for referrals to primary care physicians. Talk to friends and family about who they see and whether they would recommend their physician. Find out specifically why they did or didn't like a physician, as that can offer clues as to whether or not this physician will be a good fit for you.

- It is common for hospitals to offer a referral service that can provide you with the names of staff physicians who meet certain criteria you may be seeking.

- Read online bios about the physicians who rise to the top of these searches and see who seems like a fit for you and your preferences. Once you've narrowed the list down, schedule a phone appointment with the physician who looks most promising. Many physicians are happy to have a brief, introductory phone call with prospective patients.

You can go to the following website to help you find a physician near you:
https://doctor.webmd.com/

Federally Qualified Health Centers

If you live in the U.S., you may want to consider finding a Federally Qualified Health Center if you do not have health insurance or if you have a plan with high deductibles. Federally Qualified Health Centers are community-based centers that provide comprehensive primary care services to all persons, regardless of the person's ability to pay.

To find out more about Federally Qualified Health Centers, such as eligibility and registration information, go to the following website:
https://www.hrsa.gov/opa/eligibility-and-registration/health-centers/fqhc/index.html

You can also go to the website below to help you find a Federally Qualified Health Center in the U.S. near you: https://findahealthcenter.hrsa.gov/

POTENTIAL SOURCES FOR ASSISTANCE WITH COLORECTAL CANCER SCREENINGS

Listed below are the names and contact information for national organizations that can sometimes assist you with the cost of a colorectal cancer screening test.

COLORECTAL CANCER ALLIANCE
(877) 422-2030
Website: https://www.ccalliance.org/patient-family-support/financial-assistance-programs

Certified patient and family support navigators can help you access low or no-cost colorectal cancer screenings. They can also chat with you about a dedicated screening fund to pay for colorectal cancer screenings for black individuals in need.

COLONOSCOPY ASSIST
(855) 542-6566
Website: https://colonoscopyassist.com/Program_Details.html

This is a resource to help uninsured and self-pay individuals by providing screening tests at discounted rates.

NATIONAL CANCER INSTITUTE (NCI): CANCER INFORMATION SERVICE
(800) 422-6237
Website: www.cancer.gov

Information specialists can answer questions about cancer and provide materials. The NCI website provides comprehensive information on cancer prevention, diagnosis, treatment, statistics, research, clinical trials and news.

AMERICAN CANCER SOCIETY (ACS)
(800) ACS-2345 (800-227-2345)
(866) 228-4327
Website: http://www.cancer.org/docroot/home/index.asp?level=0

The ACS is the nationwide, community-based, voluntary health organization dedicated to eliminating cancer as a major health problem by preventing cancer, saving lives, and diminishing suffering from cancer, through research, education, advocacy, and service.

HEALTH RESOURCES AND SERVICES ADMINISTRATION (HRSA)
(888) ASK-HRSA (888-275-4772)
TTY: (888) 489-4722
Website: http://hrsa.gov/
Find a health center near you: http://findahealthcenter.hrsa.gov/

HRSA is an agency of the U.S. Department of Health and Human Services. It is the primary Federal agency for improving access to health care services for people who are uninsured, isolated or medically vulnerable.

TEMPLATE INSURANCE LETTER

The following template letter can be used, if needed, to obtain approval for coverage of colorectal cancer screening test from an insurance company.

DATE

INSURANCE COMPANY NAME
INSURANCE COMPANY ADDRESS

RE: Patient name
Member ID: Patient DOB

Dear insurance company,

Ms. [NAME] is a [AGE] year-old survivor of [CANCER]. As part of his/her therapy, he/she was treated with total body irradiation and/or radiation to the pelvis, abdomen or spine. People who have been treated with radiation to these sites are more likely to develop colorectal cancer as compared to the general population, with their elevated risk evident by the age of 30 years.

The Children's Oncology Group (www.survivorshipguidelines.org) recommends that people treated with total body irradiation and/or radiation to the pelvis, abdomen or spine have a colorectal cancer screening test starting at age 30. These people should have a colonoscopy every 5 years or a cologuard every 3 years.

These guidelines are considered to be the standard of care in North America for following cancer survivors who are high-risk for developing colorectal cancer.

In compliance with these recommendations, Mr./Ms. [NAME] requires a colonoscopy or cologuard test. If possible, please be sure to bill this as a **ROUTINE** screening test.

CPT code for colonoscopy: 45378
Billable ICD-10 codes:

- Z85.9 - Personal history of malignant neoplasm, unspecified
- Z92.3 - Personal history of radiation
- Z12.11 - Encounter for other screening for malignant neoplasm of the colon

CPT code for cologuard test: 81528
Billable ICD-10 codes:

- Z85.9 - Personal history of malignant neoplasm, unspecified
- Z92.3 - Personal history of radiation
- Z12.11 - Encounter for other screening for malignant neoplasm of the colon

I appreciate your assistance in the pre-authorization. Please let me know if you have any questions or need any further information.

Sincerely,

PHYSICIAN NAME
